# 2,6-Dichloro­aniline–4-(2,6-dichloro­anilino)­pent-3-en-2-one (1/2)

**DOI:** 10.1107/S1600536812049227

**Published:** 2012-12-08

**Authors:** Gertruida J. S. Venter, Gideon Steyl, Andreas Roodt

**Affiliations:** a Department of Chemistry, University of the Free State, PO Box 339, Bloemfontein, 9300, South Africa

## Abstract

The asymmetric unit of the title compound, C_6_H_5_Cl_2_N·2C_11_H_11_Cl_2_NO, is composed of one mol­ecule of an enamino–ketone [*i.e.* –(2,6-dichloro­phenyl­amino)­pent-3-en-2-one] and half a mol­ecule of 2,6-dichloro­aniline, the whole mol­ecule of the latter component being generated by twofold rotational symmetry. In this latter mol­ecule, there are two intra­molecular N—H⋯Cl contacts. In the enamino–ketone mol­ecule, there is an N—H⋯O hydrogen bond of moderate strength, and the dihedral angle between the benzene ring and penta­none fragment [C—C(—N)=C—C(=O)—C; planar within 0.005 (1) Å] is 81.85 (7)°. In the crystal, two mol­ecules of the enamino–ketone are bridged by a mol­ecule of 2,6-dichloro­aniline *via* N—H⋯O hydrogen bonds of moderate strength. There are also π–π inter­actions present, involving the benzene rings of inversion-related enamino–ketone mol­ecules [centroid–centroid distance = 3.724 (4) Å].

## Related literature

For the properties of enamino–ketones as liquid crystals, see: Pyżuk *et al.* (1993 ▶). For fluorescence studies of enamino–ketones, see: Xia *et al.* (2008 ▶). For the use of enamino–ketones in medicine, see: Tan *et al.* (2008 ▶); and in catalysis, see: Roodt & Steyn (2000 ▶); Brink *et al.* (2010 ▶). For background to the ligand preparation, see: Shaheen *et al.* (2006 ▶); Venter *et al.* (2010 ▶); Venter, Brink *et al.* (2012 ▶). For applications of rhodium compounds containing bidentate ligand systems, see: Pyżuk *et al.* (1993 ▶); Tan *et al.* (2008 ▶); Xia *et al.* (2008 ▶). For related rhodium enamino–­ketonato complexes, see: Brink *et al.* (2010 ▶); Damoense *et al.* (1994 ▶); Roodt & Steyn (2000 ▶); Venter, Steyl *et al.* (2012 ▶). For classification of hydrogen bonds, see: Gilli & Gilli (2009 ▶).
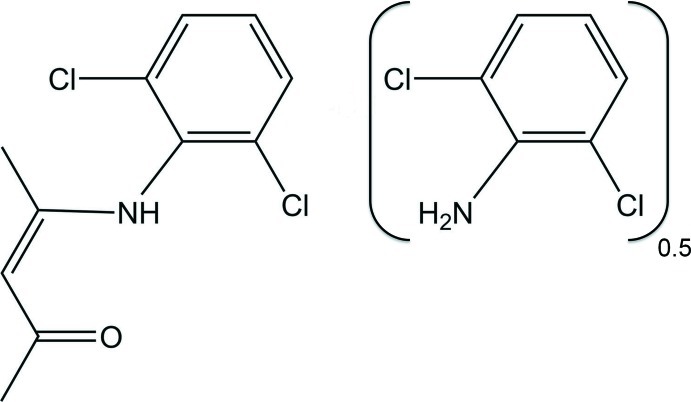


## Experimental

### 

#### Crystal data

C_6_H_5_Cl_2_N·2C_11_H_11_Cl_2_NO
*M* *_r_* = 650.23
Monoclinic, 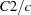

*a* = 15.7140 (1) Å
*b* = 8.7210 (2) Å
*c* = 22.9950 (4) Å
β = 104.794 (1)°
*V* = 3046.81 (9) Å^3^
*Z* = 4
Mo *K*α radiation
μ = 0.60 mm^−1^
*T* = 100 K
0.31 × 0.25 × 0.19 mm

#### Data collection

Bruker X8 APEXII 4K KappaCCD diffractometer
Absorption correction: multi-scan (*SADABS*; Bruker, 2004 ▶) *T*_min_ = 0.837, *T*_max_ = 0.895
34421 measured reflections
3785 independent reflections
3305 reflections with *I* > 2σ(*I*)
*R*_int_ = 0.034

#### Refinement

*R*[*F* ^2^ > 2σ(*F* ^2^)] = 0.027
*wR*(*F* ^2^) = 0.077
*S* = 1.04
3785 reflections
186 parameters
H atoms treated by a mixture of independent and constrained refinement
Δρ_max_ = 0.32 e Å^−3^
Δρ_min_ = −0.23 e Å^−3^

### 

Data collection: *APEX2* (Bruker, 2005 ▶); cell refinement: *SAINT-Plus* (Bruker, 2004 ▶); data reduction: *SAINT-Plus*; program(s) used to solve structure: *SHELXS97* (Sheldrick, 2008 ▶); program(s) used to refine structure: *SHELXL97* (Sheldrick, 2008 ▶); molecular graphics: *DIAMOND* (Brandenburg & Putz, 2005 ▶); software used to prepare material for publication: *WinGX* (Farrugia, 2012 ▶).

## Supplementary Material

Crystal structure: contains datablock(s) global, I. DOI: 10.1107/S1600536812049227/fb2272sup1.cif

Structure factors: contains datablock(s) I. DOI: 10.1107/S1600536812049227/fb2272Isup2.hkl

Supplementary material file. DOI: 10.1107/S1600536812049227/fb2272Isup3.cml

Additional supplementary materials:  crystallographic information; 3D view; checkCIF report

## Figures and Tables

**Table 1:** Hydrogen-bond geometry (Å, °)

*D*—H⋯*A*	*D*—H	H⋯*A*	*D*⋯*A*	*D*—H⋯*A*
N21—H21⋯Cl22	0.850 (17)	2.578 (17)	2.9710 (7)	109.4 (13)
N11—H11⋯O12	0.825 (16)	1.932 (16)	2.6223 (13)	140.7 (14)
N21—H21⋯O12	0.850 (17)	2.167 (17)	2.9732 (13)	158.4 (16)

**Fig. 1.**
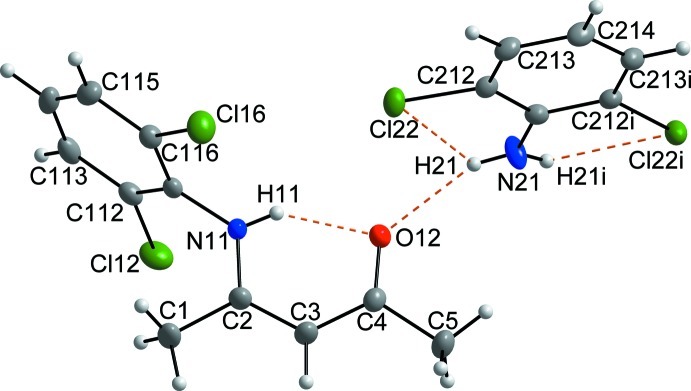
The molecular structure of the title compound, with the atom numbering. The displacement ellipsoids are drawn at the 50% probability level [Symmetry code: (i) -x, y, -z + 1/2]. The various intramolecular hydrogen bonds and contacts are shown as dashed lines.

**Fig. 2.**
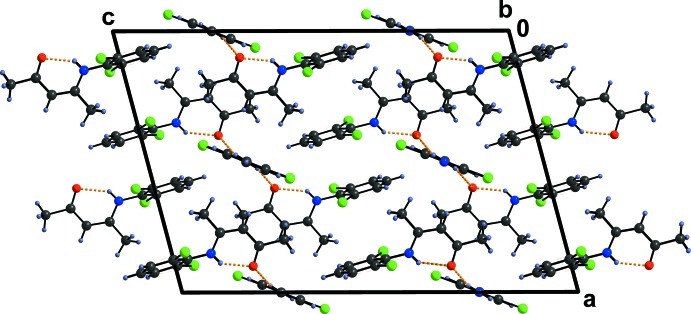
A view of the unit cell along the b axis of the crystal structure of the title compound, illustrating the intra- and intermolecular N—H···O hydrogen bonds (dashed yellow lines).

## supplementary crystallographic information

### Comment

The β-diketone compound AcacH (acetylacetone; or acetylacetonato if it is coordinated) has been studied extensively, and a large number of its derivatives have been synthesized up to date. One of these derivative types is known as enamino–ketones, which contain an unsaturated C═C bond as well as nitrogen and oxygen atoms. enamino–ketones are of interest in various fields including liquid crystals (Pyżuk *et al.*, 1993), fluorescence studies (Xia *et al.*, 2008), medicine (Tan *et al.*, 2008) and catalysis (Roodt & Steyn, 2000; Brink *et al.*, 2010).

The title enamino–ketone (Fig. 1) is a derivative of 4-(phenylamino)pent-3-en-2-one (PhonyH; Shaheen *et al.*, 2006). The 2,6-dichloroaniline molecule is located on a two-fold rotation axis. The pertinent two-fold axis passes through atoms N21, C211, C214 and H214. The molecule thus has symmetry of point group 2. The dihedral angle between the phenyl ring and the mean plane of the pentanone fragment [C1-C2(-N11)═ C3-C4(═O12)-C5; maximum deviation 0.005 (1) Å for atom C4] in the title enamino–ketone, where the substituents are situated in the *ortho* position on the phenyl ring, is 81.85 (7) °. This angle is dependent on the position of the substituent on the phenyl ring, for example compounds with *para* substituents usually display smaller dihedral angles (Venter *et al.*, 2010).

In the enamino–ketone molecule there is an intramolecular hydrogen-bond (Table 1 and Fig. 1) of moderate strength (Gilli & Gilli, 2009) in which the N_secondary amine_—H···O_keto_ is involved. There are two intramolecular contacts, N_primary amine_—H···Cl, observed in the 2,6-dichloroaniline molecule (Table 1 and Fig. 1).

In the crystal, two molecules of 4-(2,6-dichlorophenylamino)-pent-3-en-2-one and one molecule of 2,6-dichloroaniline are linked by N_primary amine_—H···O_keto_ intermolecular hydrogen bonds of moderate strength (Table 1 and Fig. 1). There are also π-π interactions present between the phenyl rings of the inversion-related 4-(2,6-dichlorophenylamino)-pent-3-en-2-one molecules, with a distance of 3.724 (4) Å between their centroids [symmetry operation: -*x*, -*y*+2, -*z*]. The packing style resulting from the respective interactions, with clearly visible π-π-stacking, is illustrated in Fig. 2.

As expected the bond distances in the title enamino–ketone differ significantly from the respective distances in compounds where the enamino–ketone is coordinated to rhodium (Venter, Steyl *et al.*, 2012; Damoense *et al.*, 1994; see Table 2), but they display similar bond distances to those observed in analogous enamino–ketones (Venter *et al.*, 2010; Venter, Brink *et al.*, 2012). The difference between the C2–C3 bond distance [1.376 (2) Å] and the C3–C4 bond distance [1.457 (3) °] indicates an unsaturated C2═C3 bond in the pentenone backbone, which is consistent with the definition of an enamino–ketone. The N11···O12 distance is longer by *ca.* 0.2 Å upon coordination when comparing the title structure and selected compounds (II) and (III) with complexes (IV) and (V), as indicated in Table 2.

### Experimental

A solution of acetylacetone (11.07 g, 0.1106 mol), 2,6-dichloroaniline (16.25 g, 0.1001 mol) and 2 drops of H_2_SO_4_(conc.) in 150 ml benzene was refluxed for 24 h in a Dean-Stark trap. The mixture was then filtered and left to crystallize by slow evaporation of the solvent yielding 23.53 g (72.37%) of cuboid crystals with lengths of up to 4 mm. The title compound is stable in air and light over a period of several months.

### Refinement

All the hydrogen atoms were identified in a difference electron density map. The NH and NH_2_ H atoms were refined with U_iso_(H) = 1.2U_eq_(N). The C-bound H atoms were placed into the idealized positions and constrained to ride on their parent atoms: C—H = 0.95 and 0.98 Å for CH and CH_3_ H atoms, respectively, with *U*_iso_(H) = k × *U*_eq_(C) where k = 1.5 for CH_3_ H atoms, and = 1.2 for other H atoms. The methyl groups were refined as rigid rotors in order to fit to the electron density.

### Crystal data

**Table:**

C_6_H_5_Cl_2_N·2C_11_H_11_Cl_2_NO	*F*(000) = 1336
*M**_r_* = 650.23	*D*_x_ = 1.418 Mg m^−^^3^
Monoclinic, *C*2/*c*	Mo *K*α radiation, λ = 0.71073 Å
Hall symbol: -C 2yc	Cell parameters from 9993 reflections
*a* = 15.7140 (1) Å	θ = 2.8–28.2°
*b* = 8.7210 (2) Å	µ = 0.60 mm^−^^1^
*c* = 22.9950 (4) Å	*T* = 100 K
β = 104.794 (1)°	Cuboid, colourless
*V* = 3046.81 (9) Å^3^	0.31 × 0.25 × 0.19 mm
*Z* = 4	

### Data collection

**Table:**

Bruker X8 APEXII 4K KappaCCD diffractometer	3785 independent reflections
Radiation source: fine-focus sealed tube	3305 reflections with *I* > 2σ(*I*)
Graphite monochromator	*R*_int_ = 0.034
ω and φ scans	θ_max_ = 28.4°, θ_min_ = 3.1°
Absorption correction: multi-scan (*SADABS*; Bruker, 2004)	*h* = −20→20
*T*_min_ = 0.837, *T*_max_ = 0.895	*k* = −11→11
34421 measured reflections	*l* = −30→30

### Refinement

**Table:**

Refinement on *F*^2^	Primary atom site location: structure-invariant direct methods
Least-squares matrix: full	Secondary atom site location: difference Fourier map
*R*[*F*^2^ > 2σ(*F*^2^)] = 0.027	Hydrogen site location: difference Fourier map
*wR*(*F*^2^) = 0.077	H atoms treated by a mixture of independent and constrained refinement
*S* = 1.04	*w* = 1/[σ^2^(*F*_o_^2^) + (0.0394*P*)^2^ + 2.1199*P*] where *P* = (*F*_o_^2^ + 2*F*_c_^2^)/3
3785 reflections	(Δ/σ)_max_ = 0.002
186 parameters	Δρ_max_ = 0.32 e Å^−^^3^
0 restraints	Δρ_min_ = −0.23 e Å^−^^3^
46 constraints	

### Special details

**Table:**

Geometry. All e.s.d.'s (except the e.s.d. in the dihedral angle between two l.s. planes) are estimated using the full covariance matrix. The cell e.s.d.'s are taken into account individually in the estimation of e.s.d.'s in distances, angles and torsion angles; correlations between e.s.d.'s in cell parameters are only used when they are defined by crystal symmetry. An approximate (isotropic) treatment of cell e.s.d.'s is used for estimating e.s.d.'s involving l.s. planes.
Refinement. Refinement of *F*^2^ against ALL reflections. The weighted *R*-factor *wR* and goodness of fit *S* are based on *F*^2^, conventional *R*-factors *R* are based on *F*, with *F* set to zero for negative *F*^2^. The threshold expression of *F*^2^ > 2σ(*F*^2^) is used only for calculating *R*-factors(gt) *etc*. and is not relevant to the choice of reflections for refinement. *R*-factors based on *F*^2^ are statistically about twice as large as those based on *F*, and *R*- factors based on ALL data will be even larger.

### Fractional atomic coordinates and isotropic or equivalent isotropic displacement parameters (Å^2^)

**Table:**

	*x*	*y*	*z*	*U*_iso_*/*U*_eq_	
C1	0.29987 (9)	1.0652 (2)	0.10718 (6)	0.0321 (3)	
H1A	0.3066	0.9907	0.0768	0.048*	
H1B	0.3555	1.0741	0.1382	0.048*	
H1C	0.2839	1.1653	0.0881	0.048*	
C2	0.22871 (8)	1.01235 (15)	0.13549 (6)	0.0223 (3)	
C3	0.24331 (9)	0.99643 (17)	0.19672 (6)	0.0261 (3)	
H3	0.3002	1.0201	0.2213	0.031*	
C4	0.17787 (8)	0.94637 (14)	0.22541 (6)	0.0214 (2)	
C5	0.20270 (10)	0.9341 (2)	0.29307 (6)	0.0344 (3)	
H5A	0.2077	1.0371	0.3107	0.052*	
H5B	0.2593	0.8809	0.3065	0.052*	
H5C	0.1573	0.8763	0.3060	0.052*	
C111	0.12706 (7)	0.99074 (14)	0.03518 (5)	0.0174 (2)	
C112	0.10240 (8)	1.12965 (14)	0.00548 (6)	0.0211 (2)	
C113	0.07626 (8)	1.13817 (16)	−0.05687 (6)	0.0252 (3)	
H113	0.0594	1.2337	−0.0762	0.030*	
C114	0.07507 (8)	1.00632 (17)	−0.09046 (6)	0.0271 (3)	
H114	0.0572	1.0116	−0.1331	0.032*	
C115	0.09966 (9)	0.86664 (16)	−0.06261 (6)	0.0256 (3)	
H115	0.0991	0.7764	−0.0858	0.031*	
C116	0.12516 (8)	0.86044 (14)	−0.00031 (6)	0.0201 (2)	
C211	0.0000	0.53418 (19)	0.2500	0.0181 (3)	
C212	0.02876 (8)	0.44701 (14)	0.20698 (5)	0.0183 (2)	
C213	0.03042 (8)	0.28839 (14)	0.20718 (5)	0.0217 (3)	
H213	0.0523	0.2345	0.1781	0.026*	
C214	0.0000	0.2083 (2)	0.2500	0.0244 (4)	
H214	0.0000	0.0993	0.2500	0.029*	
N11	0.14928 (7)	0.98030 (13)	0.09874 (5)	0.0198 (2)	
H11	0.1130 (10)	0.9500 (18)	0.1164 (7)	0.024*	
N21	0.0000	0.68981 (18)	0.2500	0.0275 (4)	
H21	0.0252 (11)	0.737 (2)	0.2268 (7)	0.033*	
O12	0.10094 (6)	0.91464 (10)	0.19690 (4)	0.02136 (19)	
Cl12	0.10261 (2)	1.29453 (4)	0.047476 (17)	0.03288 (10)	
Cl16	0.15505 (2)	0.68546 (4)	0.034586 (16)	0.03104 (10)	
Cl22	0.06336 (2)	0.54706 (3)	0.151471 (13)	0.02337 (9)	

### Atomic displacement parameters (Å^2^)

**Table:**

	*U*^11^	*U*^22^	*U*^33^	*U*^12^	*U*^13^	*U*^23^
C1	0.0179 (6)	0.0563 (9)	0.0219 (6)	−0.0084 (6)	0.0044 (5)	0.0032 (6)
C2	0.0167 (6)	0.0298 (6)	0.0201 (6)	−0.0027 (5)	0.0040 (5)	0.0004 (5)
C3	0.0184 (6)	0.0399 (7)	0.0182 (6)	−0.0047 (5)	0.0016 (5)	0.0014 (5)
C4	0.0231 (6)	0.0223 (6)	0.0181 (6)	0.0001 (5)	0.0042 (5)	0.0011 (5)
C5	0.0301 (7)	0.0548 (9)	0.0176 (6)	−0.0073 (7)	0.0049 (5)	0.0035 (6)
C111	0.0136 (5)	0.0222 (6)	0.0167 (5)	−0.0025 (4)	0.0044 (4)	0.0011 (4)
C112	0.0173 (6)	0.0208 (6)	0.0260 (6)	−0.0024 (5)	0.0069 (5)	0.0012 (5)
C113	0.0172 (6)	0.0305 (7)	0.0274 (6)	−0.0005 (5)	0.0048 (5)	0.0124 (5)
C114	0.0196 (6)	0.0446 (8)	0.0169 (6)	−0.0019 (6)	0.0045 (5)	0.0051 (5)
C115	0.0233 (6)	0.0333 (7)	0.0209 (6)	−0.0025 (5)	0.0066 (5)	−0.0055 (5)
C116	0.0182 (6)	0.0208 (6)	0.0213 (6)	0.0001 (5)	0.0049 (5)	0.0024 (5)
C211	0.0154 (8)	0.0176 (8)	0.0196 (8)	0.000	0.0012 (6)	0.000
C212	0.0180 (6)	0.0196 (6)	0.0159 (5)	0.0012 (4)	0.0017 (4)	0.0016 (4)
C213	0.0246 (6)	0.0200 (6)	0.0182 (6)	0.0029 (5)	0.0013 (5)	−0.0025 (4)
C214	0.0318 (10)	0.0155 (8)	0.0228 (9)	0.000	0.0010 (7)	0.000
N11	0.0171 (5)	0.0275 (5)	0.0151 (5)	−0.0050 (4)	0.0049 (4)	0.0001 (4)
N21	0.0375 (9)	0.0165 (7)	0.0372 (9)	0.000	0.0258 (8)	0.000
O12	0.0201 (4)	0.0243 (4)	0.0197 (4)	−0.0032 (4)	0.0052 (3)	0.0020 (3)
Cl12	0.03494 (19)	0.01929 (16)	0.0452 (2)	−0.00228 (13)	0.01176 (16)	−0.00467 (13)
Cl16	0.0402 (2)	0.02055 (16)	0.03423 (19)	0.00503 (13)	0.01296 (15)	0.00368 (12)
Cl22	0.02857 (17)	0.02216 (15)	0.02189 (15)	0.00499 (11)	0.01100 (12)	0.00329 (11)

### Geometric parameters (Å, º)

**Table:**

C1—C2	1.5028 (18)	C113—C114	1.383 (2)
C1—H1A	0.9800	C113—H113	0.9500
C1—H1B	0.9800	C114—C115	1.385 (2)
C1—H1C	0.9800	C114—H114	0.9500
C2—N11	1.3458 (16)	C115—C116	1.3867 (18)
C2—C3	1.3745 (17)	C115—H115	0.9500
C3—C4	1.4248 (18)	C116—Cl16	1.7322 (12)
C3—H3	0.9500	C211—N21	1.357 (2)
C4—O12	1.2501 (15)	C211—C212^i^	1.4103 (15)
C4—C5	1.5082 (18)	C211—C212	1.4104 (15)
C5—H5A	0.9800	C212—C213	1.3835 (17)
C5—H5B	0.9800	C212—Cl22	1.7437 (12)
C5—H5C	0.9800	C213—C214	1.3880 (16)
C111—C116	1.3948 (17)	C213—H213	0.9500
C111—C112	1.3960 (17)	C214—C213^i^	1.3881 (16)
C111—N11	1.4162 (15)	C214—H214	0.9500
C112—C113	1.3889 (18)	N11—H11	0.825 (16)
C112—Cl12	1.7316 (13)	N21—H21	0.850 (17)
			
C2—C1—H1A	109.5	C114—C113—H113	120.3
C2—C1—H1B	109.5	C112—C113—H113	120.3
H1A—C1—H1B	109.5	C113—C114—C115	120.73 (12)
C2—C1—H1C	109.5	C113—C114—H114	119.6
H1A—C1—H1C	109.5	C115—C114—H114	119.6
H1B—C1—H1C	109.5	C114—C115—C116	119.02 (12)
N11—C2—C3	120.52 (12)	C114—C115—H115	120.5
N11—C2—C1	117.71 (11)	C116—C115—H115	120.5
C3—C2—C1	121.77 (12)	C115—C116—C111	122.00 (11)
C2—C3—C4	123.62 (12)	C115—C116—Cl16	119.05 (10)
C2—C3—H3	118.2	C111—C116—Cl16	118.95 (9)
C4—C3—H3	118.2	N21—C211—C212^i^	122.62 (7)
O12—C4—C3	122.73 (11)	N21—C211—C212	122.62 (8)
O12—C4—C5	119.08 (12)	C212^i^—C211—C212	114.76 (15)
C3—C4—C5	118.18 (11)	C213—C212—C211	123.07 (12)
C4—C5—H5A	109.5	C213—C212—Cl22	119.58 (10)
C4—C5—H5B	109.5	C211—C212—Cl22	117.35 (9)
H5A—C5—H5B	109.5	C212—C213—C214	119.74 (12)
C4—C5—H5C	109.5	C212—C213—H213	120.1
H5A—C5—H5C	109.5	C214—C213—H213	120.1
H5B—C5—H5C	109.5	C213—C214—C213^i^	119.55 (16)
C116—C111—C112	117.33 (11)	C213—C214—H214	120.2
C116—C111—N11	120.94 (11)	C213^i^—C214—H214	120.2
C112—C111—N11	121.67 (11)	C2—N11—C111	125.41 (11)
C113—C112—C111	121.56 (12)	C2—N11—H11	113.8 (11)
C113—C112—Cl12	119.29 (10)	C111—N11—H11	120.7 (11)
C111—C112—Cl12	119.15 (10)	C211—N21—H21	119.2 (12)
C114—C113—C112	119.37 (12)		
			
N11—C2—C3—C4	0.0 (2)	N11—C111—C116—C115	176.95 (11)
C1—C2—C3—C4	−179.84 (14)	C112—C111—C116—Cl16	−179.90 (9)
C2—C3—C4—O12	−1.0 (2)	N11—C111—C116—Cl16	−2.72 (16)
C2—C3—C4—C5	−179.94 (14)	N21—C211—C212—C213	178.88 (9)
C116—C111—C112—C113	0.53 (18)	C212^i^—C211—C212—C213	−1.13 (9)
N11—C111—C112—C113	−176.62 (11)	N21—C211—C212—Cl22	−1.17 (10)
C116—C111—C112—Cl12	179.39 (9)	C212^i^—C211—C212—Cl22	178.83 (10)
N11—C111—C112—Cl12	2.24 (16)	C211—C212—C213—C214	2.23 (17)
C111—C112—C113—C114	−0.40 (19)	Cl22—C212—C213—C214	−177.73 (7)
Cl12—C112—C113—C114	−179.26 (10)	C212—C213—C214—C213^i^	−1.07 (8)
C112—C113—C114—C115	−0.05 (19)	C3—C2—N11—C111	−179.21 (13)
C113—C114—C115—C116	0.35 (19)	C1—C2—N11—C111	0.7 (2)
C114—C115—C116—C111	−0.20 (19)	C116—C111—N11—C2	99.59 (15)
C114—C115—C116—Cl16	179.47 (10)	C112—C111—N11—C2	−83.36 (16)
C112—C111—C116—C115	−0.23 (18)		

Symmetry code: (i) −*x*, *y*, −*z*+1/2.

### Hydrogen-bond geometry (Å, º)

**Table:**

*D*—H···*A*	*D*—H	H···*A*	*D*···*A*	*D*—H···*A*
N21—H21···Cl22	0.850 (17)	2.578 (17)	2.9710 (7)	109.4 (13)
N11—H11···O12	0.825 (16)	1.932 (16)	2.6223 (13)	140.7 (14)
N21—H21···O12	0.850 (17)	2.167 (17)	2.9732 (13)	158.4 (16)

### Table 2. Comparative geometrical parameters (Å, °) for free N,O-bidendate (N,O-bid) compounds and coordinated dicarbonyl N,O-bidendate rhodium [Rh(N,O-bid)(CO)_2_] complexes.

**Table:**

Parameters	I	II	III	IV	V
N_11_-C_111_	1.416 (2)	1.408 (2)	1.417 (2)	1.451 (2)	-
N_11_-C_2_	1.346 (2)	1.347 (2)	1.348 (1)	1.324 (2)	1.303 (6)
O_12_-C_4_	1.250 (1)	1.249 (2)	1.253 (1)	1.295 (2)	1.281 (6)
C_2_-C_3_	1.375 (2)	1.379 (2)	1.384 (2)	1.419 (3)	1.396 (7)
C_3_-C_4_	1.425 (2)	1.428 (2)	1.424 (2)	1.378 (3)	1.388 (8)
N_11_···O_12_	2.622 (2)	2.633 (2)	2.646 (1)	2.890 (2)	2.826 (6)
N_11_-C_2_···C_4_-O_12_	-0.8 (1)	5.5 (1)	1.70 (9)	1.6 (2)	1.2 (4)
Dihedral angle *^a^*	81.87 (5)	46.52 (5)	29.90 (3)	89.82 (6)	-

(I) This work. (II) 4-((2-Chloro-phenyl)amino)pent-3-en-2-one (Venter, Brink *et al.*, 2012). (III) 4-(4-Methylphenylamino)pent-3-en-2-onate (Venter *et al.*, 2010). (IV) Dicarbonyl-(4-(2,6-dimethylphenylamino)pent-3-en-2-onato- κ^2^*N*,*O*)-rhodium(I) (Venter, Steyl *et al.*, 2012). (V) (2-Imino-4-pentanonato-κ^2^*N*,*O*) -carbonyl-triphenylphosphine-rhodium(I) (Damoense *et al.*, 1994)]. *a)* The torsion angle between the phenyl ring and pentenone fragments.
